# Quorum Sensing in Some Representative Species of *Halomonadaceae*

**DOI:** 10.3390/life3010260

**Published:** 2013-03-05

**Authors:** Ali Tahrioui, Melanie Schwab, Emilia Quesada, Inmaculada Llamas

**Affiliations:** 1 Department of Microbiology, Faculty of Pharmacy, University of Granada, Campus Universitario de Cartuja, 18071 Granada, Spain; E-Mails: atahrioui@ugr.es (A.T.); melanieschwab@gmx.ch (M.S.); equesada@ugr.es (E.Q.); 2 Biotechnology Research Institute, Polígono Universitario de Fuentenueva, University of Granada, 18071 Granada, Spain

**Keywords:** hypersaline environments, halophiles, quorum sensing, *N*-acyl homoserine lactones, *Halomonadaceae*

## Abstract

Cell-to-cell communication, or quorum-sensing (QS), systems are employed by bacteria for promoting collective behaviour within a population. An analysis to detect QS signal molecules in 43 species of the *Halomonadaceae* family revealed that they produced *N*-acyl homoserine lactones (AHLs), which suggests that the QS system is widespread throughout this group of bacteria. Thin-layer chromatography (TLC) analysis of crude AHL extracts, using *Agrobacterium tumefaciens* NTL4 (pZLR4) as biosensor strain, resulted in different profiles, which were not related to the various habitats of the species in question. To confirm AHL production in the *Halomonadaceae* species, PCR and DNA sequencing approaches were used to study the distribution of the *luxI*-type synthase gene. Phylogenetic analysis using sequence data revealed that 29 of the species studied contained a LuxI homolog. Phylogenetic analysis showed that sequences from *Halomonadaceae* species grouped together and were distinct from other members of the *Gammaproteobacteria* and also from species belonging to the *Alphaproteobacteria* and *Betaproteobacteria.*

## 1. Introduction

The definition of extreme environments has been the subject of considerable controversy and as yet, due to its complexity, no consensus of opinion has been reached. In 1979 Brock defined extreme environments as those habitats with low species diversity [1]. It has been suggested, for example, that in hypersaline environments, a typical extreme habitat, environmental factors such as a high salt concentration along with low oxygen concentrations, high or low temperatures, basic pH and solar radiation may contribute to limiting their biodiversity [2]. Nevertheless, the results of a large number of ecological studies conducted recently in extreme environments indicate that in fact they contain a fairly high diversity of species [3,4,5,6].

According to 16S rDNA gene-sequence analysis, the family *Halomonadaceae* [7], within the order *Oceanospirillales,* forms a separate phylogenetic lineage within the class *Gammaproteobacteria*. *Halomonadaceae* contains the largest number of halophilic species described to date [8], including 10 genera of halophilic and halotolerant bacteria [9]: *Aidingimonas* (one species), *Carnimonas* (one species), *Chromohalobacter* (nine species), *Cobetia* (two species), *Halomonas* (80 species), *Kushneria* (five species), *Modicisalibacter* (one species) and *Salinicola* (three species); as well as two genera of non-halophilic bacteria: *Halotalea* (one species) and *Zymobacter* (one species). Many members of the *Halomonadaceae* family are moderately halophilic since they grow best in media containing from 0.5 M to 2.5 M NaCl [10], although some can grow over a very broad range of salt concentrations due to their ability to accumulate organic compounds to adapt themselves to changes in environmental osmolarity. Compatible solutes such as betaine can be taken up from the external medium or others, such as ectoine, synthesized by the cells themselves [8,11,12]. *Halomonadaceae* species have been isolated from very different habitats, including the sea, salterns, saline soils and endorheic lakes, and have also been found in some seafoods, marine invertebrates and even in a mural painting [9,13].

Extensive studies carried out over the last 30 years into this group of halophiles have led us to a better understanding of their biodiversity, phylogenetic relationships, physiological and haloadaptative mechanisms and, more recently, their biotechnological applications. Some moderate halophiles are recognised for their potential use in biotechnology because of their capacity to produce exopolysaccharides, enzymes and compatible solutes such as ectoine—used as a stabilizer for enzymes—as well as for their active role in the process of denitrification and the degradation of aromatic compounds [6,13,14].

Within the *Halomonadaceae*, *Halomonas* is one of the genera most frequently isolated from hypersaline waters and soils by conventional-culture techniques [11,15]. Molecular-ecology techniques based on 16S rDNA sequence analysis suggest that *Halomonas ventosae* is the predominant species in these habitats [16]. Nevertheless, the ecological role that *Halomonas* species play in these habitats and their relationships with other halophilic and non-halophilic microorganisms are still unknown.

Bacteria have evolved sophisticated mechanisms to co-ordinate gene expression, such as quorum sensing (QS) [17,18,19], which involves the production of signal molecules known as autoinducers. Autoinducers include, among others, *N*-acyl homoserine lactones (AHLs), produced by the *Proteobacteria*, oligopeptides, produced by the *Firmicutes*, and furanosylborate diester (AI-2), which is produced by both *Proteobacteria* and *Firmicutes* and used for interspecies communication [20,21]. The classical AHL-based systems contain a *luxI* homologue gene, which is responsible for the synthesis of AHLs, and a *luxR* homologue gene, which is an AHL-dependent transcriptional regulator. AHL-based systems involve the accumulation of AHL molecules in the extracellular medium until a critical concentration is reached, at which point the AHLs bind a transcriptional activator, which triggers the expression of target genes, including the *luxI* gene, leading in turn to the production of more AHLs [22,23,24] and the expression of virulence factors and exoenzymes, conjugal DNA transfer, control of plasmid-copy number, production of and susceptibility to antibiotics, biofilm formation and exopolysaccharide production [20,21].

AHL-dependent systems have been reported in many genera belonging to the phylum *Proteobacteria*. In addition, studies based on genome sequencing have revealed that many bacteria contain possible *luxI/luxR* homologues and, in some cases, multiple coexisting QS systems [25]. Nevertheless, little is known about the QS systems in halophilic microorganisms. In our experiments we have found that four exopolysaccharide-producing species of the genus *Halomonas* produce AHL-autoinducer molecules [26]. We have also described the structure of AHL molecules produced by *Halomonas anticariensis* (C_4_-HSL, C_6_-HSL, C_8_-HSL and C_12_-HSL) [26] and identified and characterized the QS genes *hanR/hanI* involved in their production [27]. In addition, we have proved that the QS system is regulated by a GacS/GacA two-component system, suggesting its integral involvement in the intercellular communication strategies of this bacterium [28].

In this present study we have detected and identified quorum-sensing systems that rely upon the production of AHLs in 43 species belonging to the *Halomonadaceae* family. Using PCR and DNA sequencing approaches, we have studied the distribution of the LuxI-type synthase in 29 of these species and constructed a phylogenetic tree, that was based on a partial sequence of this protein.

## 2. Results and Discussion

### 2.1. Detection of AHLs in the Halomonadaceae Family

We have investigated the existence of AHL-dependent QS systems in 43 species of the *Halomonadaceae* family. These 43 bacteria, the type strains of their respective species, were isolated from very different saline habitats, including salterns, saline soils, marshes and seawater, among others (Table 1). They include 12 species discovered by our research group during the course of ecological and taxonomic studies conducted in hypersaline environments in Spain, Chile and Morocco [29,30,31,32,33,34,35,36,37,38,39,40,41]. The strains used in this study include representatives of the following genera: *Chromohalobacter* (one species), *Cobetia* (one species), *Halomonas* (33 species), *Halotalea* (one species), *Kushneria* (three species), *Modicisalibacter* (one species) and *Salinicola* (two species). So far AHL signal molecules have only been detected in four exopolysaccharide-producing species of *Halomonas* described in our laboratory: *H. eurihalina, H. maura, H. ventosae* and *H. anticariensis* [26].

**Figure 1:**
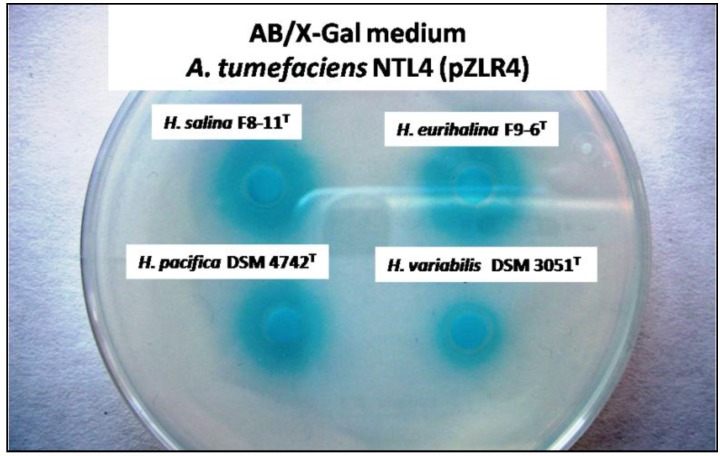
*N*-acyl homoserine lactone (AHL) production by *Halomonas salina* F8-11^T^, *H. eurihalina* F9-6^T^, *H. pacifica* DSM 4742^T^ and *H. variabilis* DSM 3051^T^. A volume of 5µL of AHLs previously extracted from the bacterial cultures were visualized on agar plate diffusion assay by means of the indicator strain *A. tumefaciens* NTL4 (pZLR4).

**Figure 2:**
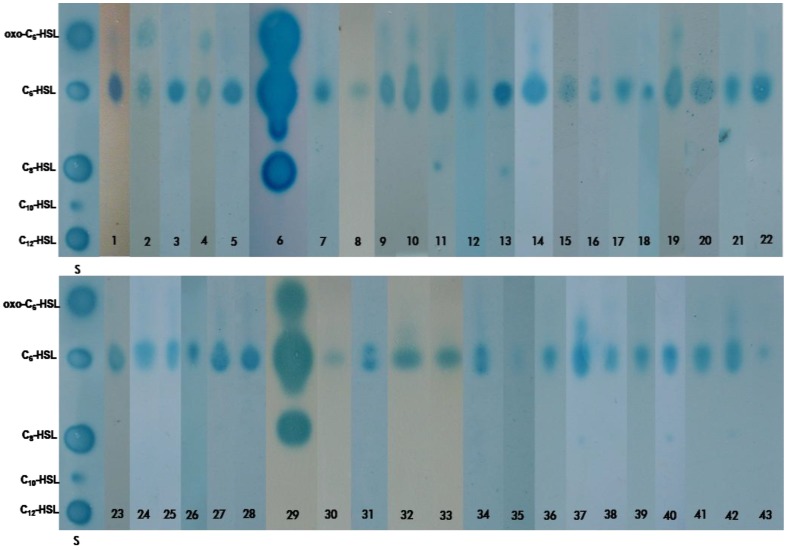
Thin-layer chromatography (TLC) analysis of the AHLs produced by the 43 species of *Halomonadaceae*: lane 1, *Carnimonas nigrificans* CTCBS1^T^, lane 2, *Chromohalobacter salexigens* DSM3043^T^; lane 3, *Cobetia marina* 219^T^; lane 4, *Halomonas alimentaria* YKJ-16^T^; lane 5, *H. almeriensis* M8^T^; lane 6, *H. anticariensis* FP35^T^; lane 7, *H. aquamarina* 558^T^; lane 8, *H. campaniensis* 5AG^T^; lane 9, *H. cerina* SP4^T^; lane 10, *H. denitrificans* M29^T^; lane 11, *H. desiderata* FB2^T^; lane 12, *H. elongata* 1H9^T^; lane 13, *H. eurihalina* F9-6^T^, lane 14, *H. fontilapidosi* 5CR^T^, lane 15, *H. gudaonensis* SL014B-69^T^; lane 16, *H. halmophila* ACAM 71^T^; lane 17, *H. halodenitrificans* ATCC 13511^T^; lane 18, *H. halodurans* DSM 5160^T^; lane 19, *H. koreensis* SS20^T^; lane 20, *H. magadiensis* 21 MI^T^; lane 21, *H. maura* S-31^T^; lane 22, *H. meridiana* ACAM 246^T^; lane 23, *H. mongoliensis* Z-7009^T^; lane 24, *H. nitroreducens* 11-S^T^; lane 25, *H. organivorans* G-16.1^T^; lane 26, *H. pacifica* DSM 4742^T^; lane 27, *H. pantelleriensis* AAP^T^; lane 28, *H. ramblicola* RS-16^T^; lane 29, *H. rifensis* HK31^T^; lane 30, *H. saccharevitans* AJ275^T^; lane 31, *H. salina* F8-11^T^; lane 32, *H. shengliensis* SL014B-85^T^; lane 33, *H. stenophila* N12^T^; lane 34, *H. subglaciescola* ACAM 12^T^; lane 35, *H. variabilis* DSM 3051^T^; lane 36, *H. ventosae* Al-12^T^, lane 37, *Halotalea alkalilenta* AW-7^T^; lane 38, *Kushneria avicenniae* MW2a^T^; lane 39, *K. indalinina* CG2.1^T^; lane 40, *K. marisflavi* SW32^T^; lane 41, *Modicisalibacter tunisiensis* LIT2^T^; lane 42, *Salinicola halophilus* CG4.1^T^; lane 43, *S. salarius* M27^T^. Each lane contains 10 µL of AHL crude extract except for lanes 6 and 29, which contain 5 µL. Lane S; synthetic AHL standards: oxo-C_6_-HSL (4.7 pmol), C_6_-HSL (804 pmol), C_8_-HSL (31.6 pmol), C_10_-HSL (2 nmol), C_12_-HSL (4.8 nmol).

To overcome the limitation of the “cross-streak” method [42], in which each couple of sensor test strains must be cultured under optimum conditions without interfering with each other, we extracted AHLs from all the strains assayed and added them to agar plates upon which the biosensor strain had already been spread (see Experimental Section). Our choice of biosensor strains was ultimately based on previous experience in our laboratory [26]. Thus we chose the *Chromobacterium violaceum* strain CV026, a mutant which cannot synthesize its own quorum-sensing signal molecules and responds to exogenously added short-chain AHLs (C_4_-C_6_-HSLs), producing a pigment called violacein [43], and also *Agrobacterium tumefaciens* NTL4 (pZLR4), a sensitive, broad-spectrum AHL-responsive reporter that is unable to produce its own AHLs and contains a *lacZ* fusion to the quorum-sensing regulated gene *traG*. This latter strain is sensitive to AHLs with medium-to-long acyl chains that, when added exogenously, activate *lacZ* fusion, which is detectable by the appearance of a blue stain in the presence of X-Gal [44]. All the strains tested synthesized signal molecules to activate the biosensor *A. tumefaciens* NTL4 (pZLR4) (see Figure 1 for some examples). No signal was detected when a sample from an uninoculated cultured medium of MY 7.5% (w/v) was tested as negative control (data not shown). These results initially suggested that most strains were able to produce AHLs and therefore probably possess at least one AHL-QS system. Nevertheless, only *Halomonas rifensis* HK31^T^ and *H. anticariensis* FP35^T^, used as control, produced AHLs in sufficient quantities to activate *C. violaceum* CV026 under our assay conditions. As is demonstrated below, these two strains produce about five times more AHL than the rest of the species tested (Figure 2). We have in fact already described how some species of *Halomonas*, such as *H. anticariensis* FP35^T^, synthesize much greater quantities of AHLs than others, such as *H. eurihalina, H. maura* and *H. ventosae* [26].

### 2.2. Characterization of the AHLs

The use of the indicator organisms in combination with thin-layer chromatography (TLC) provides a simple, rapid way of determining the number and nature of the AHLs produced by a particular strain [44]. We analysed the culture extracts of the 43 *Halomonadaceae* strains (Table 1) using TLC in combination with the biosensor *A. tumefaciens* NTL4 (pZLR4). This analysis showed the production of different AHL profiles amongst the various genera e.g. *Chromohalobacter salexigens* (Figure 2, lane 2), *Cobetia marina* (Figure 2, lane 3), *Halomonas anticariensis* (Figure 2, lane 6), * Halotalea alkalilenta* (Figure 2, lane 37), *Kushneria marisflavi* (Figure 2, lane 40) and *Salinicola halophilus* (Figure 2, lane 42) and also among certain species such as *Halomonas alimentaria*YKJ-16^T^ (Figure 2, lane 4), *H. anticariensis* FP35^T^ (Figure 2, lane 6), *H. desiderata* FB2^T^ (Figure 2, lane 11) and *H. eurihalina* F9-6^T^ (Figure 2, lane 14). Similarly, in a previous study [26] we found that strains belonging to the same species showed the same AHL profiles whilst different species showed different profiles. In just the same way, significant differences have been identified in the AHL profiles of the marine species *Vibrio salmonicida* [45] and *V. anguillarum* [46].

The AHL patterns from *Halomonadaceae* species contain from one to three spots with mobilities similar to those of the C_8_-HSL, C_6_-HSL and 3-oxo-C_6_-HSL standards. Differences were also observed in the quantities of AHLs synthesized, *Halomonas anticariensis* FP35^T^ and *Halomonas rifensis* HK31^T ^synthesizing about five times more AHLs than any of the other 41 species examined (Figure 2. lanes 6 and 29). *Halomonas variabilis* DSM 3051^T^ (Figure 2, lane 35) and *Salinicola salarius* M27^T^ (Figure 2, lane 43) produce as low an amount of signal as that from the uninoculated cultured medium MY 7.5% (w/v) [26], although, their AHL-extracts did activate the *A. tumefaciens* NTL4 (pZLR4) indicator strain when the diffusion plate assay was carried out (data not shown). The most predominant AHL molecule was C_6_-HSL. In *H. anticariensis* FP35^T^ this AHL had been previously identified by gas chromatography/mass spectrometry (GM/MS) and electrospray ionization tandem mass spectrometry (ESI MS/MS) [26], suggesting that this signal molecule may well be biologically active in intercellular communication strategies within the *Halomonadaceae* family. The synthesis of short-chain-acyl AHLs, such as C_6_-HSL and C_8_-HSL, is also very common among the species belonging to the *Vibrionaceae* family, which are ubiquitous in marine environments [45,46].

### 2.3. Distribution of the Autoinducer Synthase Gene

The *luxI* autoinducer synthase gene has been reported to be responsible for AHL production [18,47]. Therefore we tested its presence in the genome of 43 *Halomonadaceae* species, using *luxI* primers to amplify by PCR a fragment (300–400 bp) which forms part of the active site of the enzyme [48]. In this way we identified them in 29 species. On sequencing they turned out to be homologous to a *luxI* gene fragment. We did not, however, detect PCR fragments in the other 14 AHL-producing strains, possibly due to primer mismatching. In the cases of *Chromohalobacter salexigens* and *Halomonas elongata,* both of which do produce AHLs and the sequenced genomes of which are available, no *luxI* gene could be identified, due probably to their having different AHL synthases, either belonging to the LuxM protein family found in the genus *Vibrio* [49,50] or to the HdtS protein family identified in *Pseudomonas fluorescens* [51].

**Figure 3:**
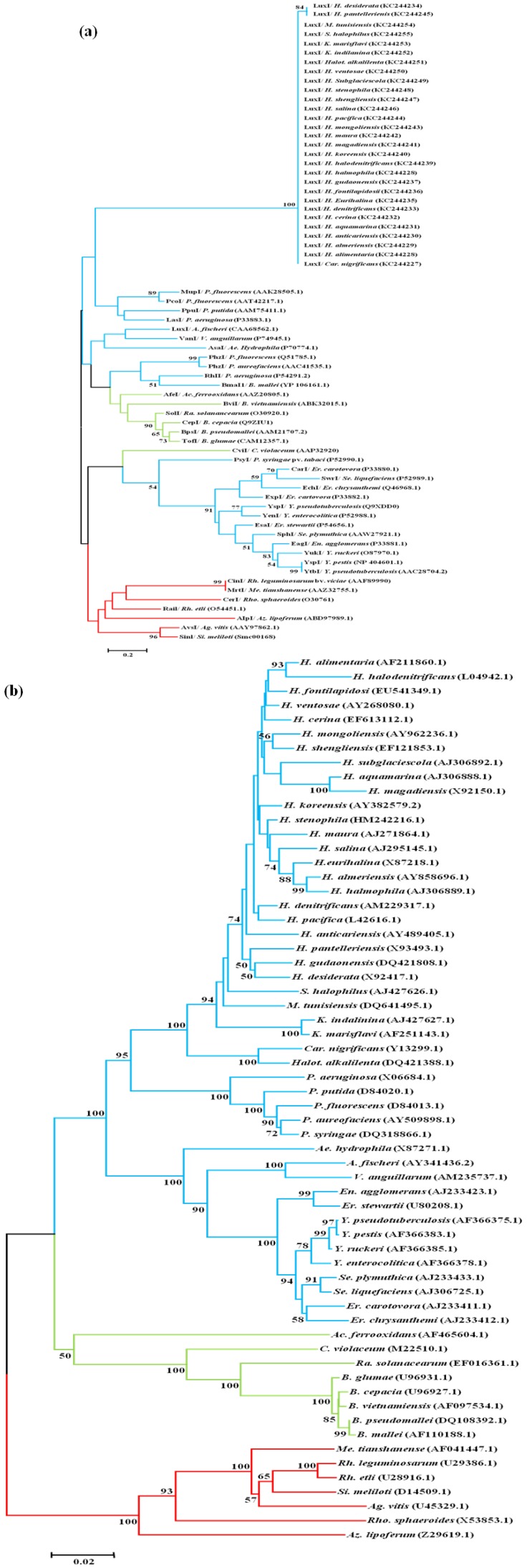
Phylogenetic trees based on LuxI sequences (a) and 16S rDNA sequences (b) found in some members of *Alphaproteobacteria*, *Betaproteobacteria* and *Gammaproteobacteria*, including the species studied here belonging to the *Halomonadaceae* family. Abbreviations for bacterial genus names: *A, Aliivibrio; Ac, Acidithiobacillus; Ae, Aeromonas; Ag, Agrobacterium; Az, Azospirillium; B, Burkholderia; Car, Carnimonas; C, Chromobacterium; Er, Erwinia; En, Enterobacter; H, Halomonas; Halot, Halotalea; K, Kushneria; M, Modicisalibacter; Me, Mesorhizobium; Ps, Pseudomonas; Rh, Rhizobium; Ra, Ralstonia; Rho, Rhodobacter; Si, Sinorhizobium; S, Salinicola; Se, Serratia; V, Vibrio; Y, Yersinia.* The scale bar indicates the mean number of substitutions per site. Bootstrap values were obtained from 1,000 replicates via neighbour-joining algorithms using the MEGA program. Only branches with values >50% are shown. The branches highlighted in red are sequences from *Alphaproteobacteria*, in green from *Betaproteobacteria* and in blue from *Gammaproteobacteria*. The sequence accession numbers are given in brackets.

To carry out a phylogenetic analysis of the LuxI synthase in the 29 positive species of the *Halomonadaceae* family we conducted a multiple sequence alignment, including amino-acid sequences of LuxI synthase that had been experimentally determined in other members of the phyla *Alphaproteobacteria, Betaproteobacteria* and *Gammaproteobacteria*. The phylogenetic tree constructed according to the neighbour-joining method showed that all the amino-acid sequences from the *Halomonadaceae* family grouped together and were distinct from the rest of the *Gammaproteobacteria* analysed, and also from the species belonging to the *Alphaproteobacteria* and *Betaproteobacteria* (Figure 3a). The clustering of the 29 *Halomonadaceae* in which the *luxI* fragment was detected was not related to the habitat from which they were isolated (Table 1). The distribution of the *Halomonadaceae* family with respect to the rest of species analysed in the phylogenetic tree based on the 16S rDNA sequences (Figure 3b) was similar to that obtained from the LuxI sequence (Figure 3a). This result indicates that the LuxI amino-acid partial region used in this study is conserved among the family *Halomonadaceae* members.

## 3. Experimental Section

**Bacterial strains.** We used the type strains of 43 species belonging to the *Halomonadaceae* family (Table 1). Strains were cultured at 32 °C in MY medium (3 g malt extract, 3 g yeast extract, 10 g glucose and 5 g peptone per litre) [52,53] modified with a balanced mixture of sea salts [54].

**Table 1:** Species of the *Halomonadaceae* family included in this study [9].

Species	Strain	Ecological Niches
1. *Carnimonas nigrificans*	CTCBS1^T^	Cured meat, Spain
2. *Chromohalobacter salexigens*	DSM 3043^T^	Solar saltern, Netherlands
3. *Cobetia marina*	219^T^	Sea water, USA
4. *Halomonas alimentaria*	YKJ-16^T^	Jeotgal, a traditional Korean fermented seafood, Korea
5. *H. almeriensis*	M8^T^	Solar saltern, south-east Spain
6. *H. anticariensis*	FP35^T^	Saline wetland, southern Spain
7. *H. aquamarina*	558^T^	Pacific ocean
8. *H. campaniensis*	5AG^T^	Mineral pool, Italy
9. *H. cerina*	SP4^T^	Saline soil, Spain
10. *H. denitrificans*	M29^T^	Saline water, Korea
11. *H. desiderata*	FB2^T^	Municipal sewage works, Germany
12. *H. elongata*	1H9^T^	Solar saltern, Netherlands
13. *H. eurihalina*	F9-6^T^	Saline soil, Spain
14. *H. fontilapidosi*	CR-5^T^	Saline soil, southern Spain
15. *H. gudaonensis*	SL014B-69^T^	Saline soil contaminated by crude oil, China
16. *H. halmophila*	ACAM 71^T^	Dead Sea, Israel
17. *H. halodenitrificans*	ATCC 13511^T^	Meat in brine
18. *H. halodurans*	DSM 5160^T^	Great Bay estuary, USA
19. *H. koreensis*	SS20^T^	Solar saltern, Korea
20. *H. magadiensis*	21 MI^T^	Soda lake, East-African Rift Valley
21. *H. maura*	S-31^T^	Saltern, Morocco
22. *H. meridiana*	ACAM 246^T^	Saline lake, Antarctic
23. *H. mongoliensis*	Z-7009^T^	Soda lake, Mongolia
24. *H. nitroreducens*	11-S^T^	Solar saltern, Chile
25. *H. organivorans*	G-16.1^T^	Hypersaline habitats contaminated by aromatic organic compounds, southern Spain
26. *H. pacifica*	DSM 4742^T ^	Pacific ocean
27. *H. pantelleriensis*	AAP^T^	Hard lake sand, Pantelleria island, Italy
28. *H. ramblicola*	RS-16^T^	Saline soil, south-east Spain
29. *H. rifensis*	HK-31^T^	Solar saltern, Morocco
30. *H. saccharevitans*	AJ275^T^	Salt lake and a subterranean saline well, China
31. *H. salina*	F8-11^T^	Saline soil, Spain
32. *H. shengliensis*	SLO14B-85^T^	Saline soil contaminated with crude oil, China
33. *H. stenophila*	N12^T^	Saline soil, Spain
34. *H. subglaciescola*	ACAM 12^T^	Antarctic hypersaline, meromictic lake
35. *H. variabilis*	DSM 3051^T ^	Great Salt Lake, USA
36. *H. ventosae*	Al-12^T^	Saline soil, south-eastern Spain
37. *Halotalea alkalilenta*	AW-7^T^	Alkaline olive-mill waste (alpechin), Greece
38. *Kushneria avicenniae*	MW2a^T^	Salty leaves of *Avicennia germnans* trees growing near solar salterns, Puerto Rico
39. *K. indalinina*	CG2.1^T^	Solar saltern, south-east Spain
40. *K. marisflavi*	SW32^T^	Water from the Yellow Sea, Korea
41. *Modicisalibacter tunisiensis*	LIT2^T^	Oilfield-water injection sample, southern Tunisia
42. *Salinicola halophilus*	CG4.1^T^	Solar saltern, south-east Spain
43. *S. salarius*	M27^T^	Saline water, Korea

Note: Species shaded in grey indicates that the LuxI homolog has been detected by PCR.

*Chromobacterium violaceum* CV026 was cultured at 30 °C in LB medium supplemented with 2.5 mM CaCl_2_ and 2.5 mM MgSO_4_ (LB/MC) and containing 50 µg kanamycin per mL [43]. *Agrobacterium tumefaciens* NTL4 (pZLR4) was cultured at 30 °C in LB medium supplemented with 2.5 mM CaCl_2_ and 2.5 mM MgSO_4_ (LB/MC), in MGM minimal medium (11 g Na_2_HPO_4_, 3 g KH_2_PO_4_, 0.5 g NaCl, 1 g glutamate, 10 g mannitol, 1 mg biotin, 27.8 mg CaCl_2_ and 246 mg MgSO_4_ per litre) containing 50 µg gentamycin per ml, and in AB medium [44,55].

*Extraction and detection of AHLs***.** AHL molecules were extracted following the technique described in our previous studies [56,57]. Briefly, 20 mL cultures were grown until the early stationary phase (optical density of approximately 2.8 at 600 nm) and then extracted twice with equal volumes of dichloromethane. The extracts were dried and suspended in 40 μL of 70% v/v methanol.

To detect AHLs, an overnight culture of one of the AHL indicator strains [*Chromobacterium violaceum* CV026 or *Agrobacterium tumefaciens* NTL4 (pZLR4)] was diluted 1:100 in 5ml of the corresponding medium and poured onto LB/MC and AB supplemented with 80 μg of 5-bromo-4-chloro-3-indolyl-β-D-galactopyranoside (X-Gal) per ml agar plates. Once the plates were dry, paper disks 5 mm in diameter were placed onto them and the AHL samples applied. The assay plates were incubated overnight at 32 °C to allow the indicator organisms to grow and surround the paper disks with either purple or blue haloes.

*Thin-layer chromatography analysis of AHLs.* To characterize the AHLs, the samples were subjected to analytical and preparative thin-layer chromatography (TLC). AHL samples and standards were spotted onto a TLC plate and developed with 70% v/v methanol in water. The plate was air-dried and overlaid with top agar containing the *A. tumefaciens* NTL4 (pZLR4) indicator strain before being incubated at 32 °C. For the *A. tumefaciens* NTL4 (pZLR4) overlay, a 6–8 h culture in MGM medium was mixed with an equal volume of fresh medium, 1.5% w/v Bacto Agar and 80 μg of X-Gal per mL [26].

The standard AHLs used were: *N*-(β-ketocaproyl)-dl-homoserine lactone (3-oxo-C_6_-HSL), *N*-hexanoyl-dl-homoserine lactone (C_6_-HSL), *N*-octanoyl-dl-homoserine lactone (C_8_-HSL) and *N*-decanoyl-dl-homoserine lactone (C_10_-HSL) (Sigma^®^).

*Chromosomal DNA extraction, autoinducer synthase gene amplification and sequencing.* Chromosomal DNA was isolated and purified according to Marmur's protocol [58], modified by Martín-Platero and co-workers [59]. The purified DNA was dissolved in 50 μL doubly distilled water and checked by agarose gel electrophoresis [60] An internal segment of the autoinducer synthase gene was amplified from approximately 100 ng of chromosomal DNA by using the primers *luxI*-F: 5'-GGGAGATATATACTGTAA-3' and *luxI*-R: 5'-TGAGGTATTATTCTGCAA-3'. These primers were designed to target a highly conserved region of the *hanI* autoinducer synthase gene of *Halomonas anticariensis* FP35^T^. The *hanI* gene is about 645 bp and the primer pair amplified approximately 386 bp of the conserved active site of the enzyme which contains the three conserved amino acids Arg71 (R71), Glu101 (E101) and Arg104 (R104) [27]. PCR entailed 30 cycles of 30 s at 95 °C, 30 s at 50 °C and 30 s at 72 °C. The annealing temperature was determined by PCR with a temperature gradient from 40 °C to 60 °C. All of the PCRs were run in a T100^TM^ thermal cycler (Bio-Rad).

The PCR fragments were purified and sequenced with *luxI*-F or *luxI*-R primers using a BigDye Terminator Cycle Sequencing Kit in an ABI 3100 DNA sequencer (Applied Biosystems). The DNA sequences thus obtained were analysed using a BLAST search of the GenBank database [61] to align homologous regions of autoinducer synthase gene sequences from different isolates.

*Phylogenetic analysis.* A phylogenetic tree was constructed using version 4 of the MEGA (Molecular Evolutionary Genetics Analysis) software [62] after multiple alignments of the data by CLUSTALW [63] and the alignments were checked manually. Distances and clustering were determined according to the neighbour-joining method and bootstrap values were measured on the basis of 1,000 replications.

*Nucleotide sequence accession number.* The autoinducer synthase DNA sequences reported here have been deposited in the GenBank database under accession numbers from KC244227 to KC244255.

## 4. Conclusions

Screening for AHL signal molecules in 43 species belonging to the *Halomonadaceae* family revealed that the AHL-QS system is widespread within this group of bacteria. We did however find diversity within the AHL-profile signalling molecules produced by the different genera, and even between the molecules produced by different species from the same genus. Such variety would seem to be consistent with the ecological, physiological, metabolic and taxonomic diversity among them. The role of QS signalling in these extremophilic microorganisms remains to be elucidated and further work needs to be done to explore this bacterial cell-cell communication process in the multispecies communities.
